# Perioperative transfer of patients with potential spinal injury: A survey-based pre- and post-intervention study of a targeted training program

**DOI:** 10.5339/qmj.2026.30

**Published:** 2026-06-10

**Authors:** Bianca Wahlen, Ayman El-Menyar, Ahammed Mekkodathil, Amal J. Alhajaji, Kristoffer John Tapic, Ma. Darly Baltazar, Misty John, Mustafa Abdel Wahed, Maila Galvez, Hassan Al-Thani

**Affiliations:** Department of Anesthesiology, Hamad Medical Corporation (HMC), Doha, Qatar; Clinical Research, Trauma Surgery, HMC, & Weill Cornell Medicine, Doha, Qatar; Clinical Research, Trauma Surgery, HMC, Doha, Qatar; Department of Operating Theatre, HMC, Doha, Qatar; Nursing and Midwifery Education Department, HMC, Doha, Qatar; Department of Surgery, Trauma Surgery, Hamad Medical Corporation, Doha, Qatar

**Keywords:** Spinal cord injuries, operating room nursing, education, patient safety, trauma care

## Abstract

**Background:**

Safe perioperative transfer of patients with potential spinal cord injury (SCI) is essential to prevent secondary neurological damage. Targeted training of operating room nurses (ORNs) is critical for ensuring adherence to best-practice transfer techniques.

**Objectives:**

To determine the effect of a scenario-based training intervention on knowledge and self-reported practices of ORNs regarding safe perioperative transfer of patients with potential SCI.

**Methods:**

This survey-based pre- and post-intervention study was conducted at Hamad General Hospital, Doha, Qatar, between January and March 2024. A structured self-administered questionnaire was administered to ORNs before and after a targeted training intervention. Variables included preferred transfer device, number of staff required, leadership during transfer, hand positioning, and command sequence. Descriptive statistics and McNemar’s test were used for analysis.

**Results:**

A total of 138 ORNs participated (mean age, 37.6 ± 6.2 years; 68% female). Use of plastic boards increased from 88% (*n* = 122) to 93% (*n* = 128) post-intervention (P < 0.05). Compliance with the recommended four-person log-roll team improved from 67% (*n* = 92) to 79% (*n* = 109). Reliance on anesthetists for transfer leadership increased from 41% to 58%, while inappropriate reliance on other staff decreased from 33% to 28%.

**Conclusion:**

A targeted scenario-based training intervention significantly improved ORNs’ knowledge and self-reported adherence to safe perioperative transfer practices for patients with potential SCI.

## 1. INTRODUCTION

In the Middle East and North Africa (MENA) region, the incidence of traumatic spinal cord injury (SCI) varies, with motor vehicle accidents being the predominant cause, followed by falls and violence.^1^ A systematic review reported that the most affected age groups are 20 to 29 and 30 to 39 years, with males accounting for approximately 77% of cases.^2^ In Saudi Arabia, high annual incidence rates of SCI were reported, which vary between 16 and 64 per 100,000 individuals.^3^ Motor vehicle accidents have been identified as the leading cause in Saudi Arabia and Qatar.^3,4^

Furthermore, a study from Qatar reported that approximately 29% of patients admitted with fall-related injuries sustained SCIs, underscoring the high risk associated with falls.^5^ Individuals with SCIs face a 2 to 5 times higher risk of mortality compared to those without such injuries, leading to substantial individual and societal costs.^2^ The in-hospital mortality rate among SCI patients in Qatar was documented at 5%, highlighting the importance of prompt evaluation and early management of SCIs.^4^

Maintaining complete spinal alignment during any movement or handling is essential when an acute SCI is suspected.^6,7^ Careful handling, positioning, and turning are necessary to prevent or significantly reduce pain, discomfort, skin damage, and the risk of secondary injuries.^8^ To ensure safe management, the implementation of evidence-based guidelines was necessary in both prehospital and in-hospital care settings.^9,10,11,12^ At the time of the study, limited evidence existed regarding best practices for transferring patients within the operating theater, and no formal guidelines existed in our institution. This highlights the need for structured training programs to improve adherence to safe patient transfer practices among operating room (OR) personnel. The primary objective of this study was to determine the effect of a scenario-based training intervention on the knowledge and self-reported practices of operating room nurses (ORNs) regarding the safe perioperative transfer of patients with potential SCI.

## 2. METHODOLOGY

This study employed a prospective survey-based pre- and post-intervention design and was conducted among ORNs of Hamad General Hospital, Doha, Qatar, between January and March 2024. A structured self-administered questionnaire was developed following an extensive literature review on SCI transfer techniques.^13^ Content validity was established through expert review involving ORN educators and anesthetists. The questionnaire was pilot tested among 10 ORNs to ensure clarity and relevance before final implementation. The structured questionnaire included 12 items assessing demographics (*n* = 4) and knowledge and practices (KAP) related to patient transfer (*n* = 8). The survey was administered in English before and after the intervention. To ensure confidentiality, each participant was assigned a unique subject code for data recording.

### 2.1 Inclusion criteria

- ORNs who were actively involved in perioperative patient transfers.
- Nurses who provided verbal consent to participate in the study.

### 2.2 Exclusion criteria

- Nurses who were not working in the operating theatre.
- Nurses who declined participation.

For this study, “potential SCI” was defined as patients with suspected spinal injury, unstable spinal fractures, or spinal injuries that had not yet been clinically or radiologically cleared at the time of perioperative transfer. Baseline data were collected using a validated questionnaire that assessed demographic variables and KAP related to patient transfer, including preferred transfer device, number of staff required, leadership during transfer, hand positioning, and command sequence.

The intervention consisted of scenario-based training sessions conducted using mannequins, trolleys, plastic boards, and plastic sheets.^13^ Each session lasted 10 to 15 minutes and focused on maintaining spinal alignment, enhancing team coordination, and reinforcing appropriate leadership and command sequence during patient transfers. Anesthetists and nurse educators supervised training.

Following completion of the training intervention, the same questionnaire was administered immediately as a post-intervention survey to evaluate changes in knowledge and self-reported practices. The sample size was determined using a convenience sampling approach, in which all eligible ORNs available during the study period were invited to participate, as this was a pragmatic educational intervention study without a predefined effect size to inform formal sample size calculation. While this method allowed for the inclusion of a broad group of participants, it may limit the power of the findings. Future research could benefit from a larger, confirmatory study using a statistically driven sampling strategy to enhance the robustness and generalizability of the results. A total of 138 ORNs participated in the study, selected using convenience sampling of available staff during the study period. This study was approved by the Institutional Review Board of our institution (MRC# 01-22-473/amendment 02 on December 9, 2023).

### 2.3 Statistical analysis

Data were analyzed using SPSS software (version 25; SPSS Inc.). Descriptive and inferential statistical methods were applied. Continuous variables are presented as means with standard deviations (SD) or as medians with ranges, while categorical variables are expressed as frequencies and percentages. Paired pre- and post-intervention responses were compared using McNemar’s test where appropriate. Statistical assumptions for McNemar’s test, including expected frequencies, were checked and met, ensuring validity for analysis. A P-value of less than 0.05 was considered statistically significant.

## 3. RESULTS

A total of 138 ORNs participated in the survey, with a mean age of 37.6 ± 6.2 years. Among the respondents, 68% (*n* = 94) were female, while 32% (*n* = 44) were male. The median OR experience among the nurses was 10 years, ranging from 1 month to 30 years (Table 1).

Before the intervention, 65% (*n* = 90) of respondents thought there was an institutional policy regarding the transfer of patients with potential spinal injuries, but this perception dropped to 50% (*n* = 68) post-intervention (P = 0.001). Regarding self-awareness of patient injuries, 92% (*n* = 127) of participants reported checking before performing patient transfers, which increased to 95% (*n* = 131) following the intervention (P = 0.001; Table 2).

Since the question involves more than two response categories, McNemar’s test for paired responses is used.

In the pre-intervention survey, 41% (*n* = 57) of ORNs reported consulting anesthetists on patient transfer, while 33% (*n* = 46) relied on any staff nurse, surgeon, or anesthetist. Post-intervention, reliance on anesthetists increased to 58% (*n* = 80), while the proportion of consulting staff nurses, surgeons, or anesthetists decreased to 28% (*n* = 39; P = 0.001). When asked about the preferred tool for transferring patients with potential spinal injuries from a trolley to an operating table, 122 nurses (88%) initially responded “plastic board,” and this preference increased significantly to 93% (*n* = 129) post-intervention (P = 0.001).

**Figure 1.**
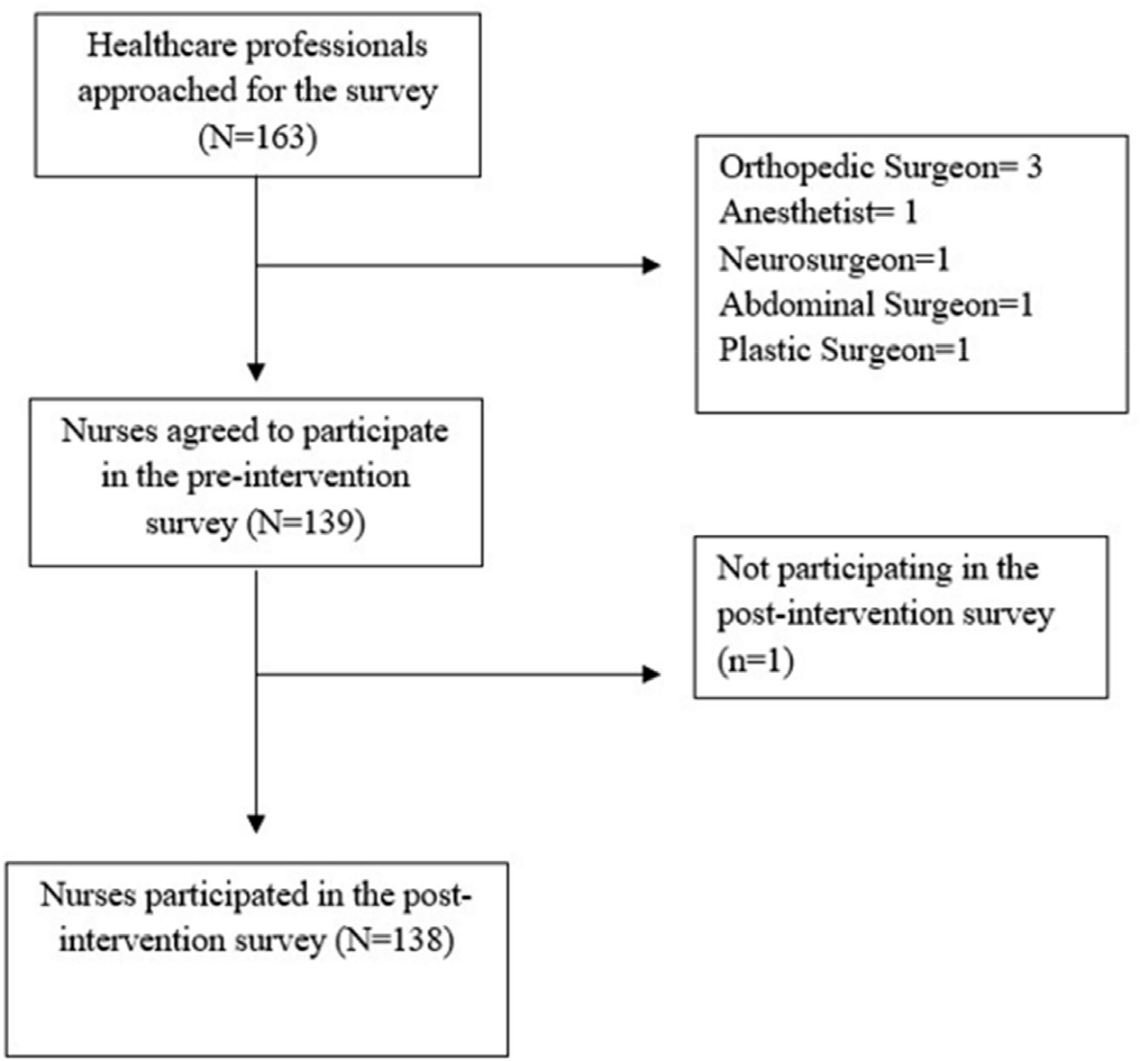
Flowchart of perioperative transfer procedures for patients with potential spinal injury.

Regarding the minimum number of staff required to transfer a patient with a normal body mass index and potential spinal injury, 67% (*n* = 92) of the nurses in the pre-intervention survey stated that four staff members were necessary. This figure increased to 79% (*n* = 109) post-intervention (P = 0.001). The importance of hand positioning in log rolling was affirmed by 98% (*n* = 135) of the nurses in the pre-intervention survey, which increased slightly to 99% (*n* = 137) post-intervention (Table 2; Figure 1).

When asked about the number of commands given during patient transfers, most pre-intervention responses (*n* = 67; 49%) indicated three commands were required. This remained consistent in the post-intervention survey, with 66 respondents giving the same answer. Both pre- and post-intervention surveys showed that most nurses believed anesthetists were responsible for issuing commands to initiate patient transfers from a trolley to an operating table. This perception was reported by 73% (*n* = 101) during the pre-intervention survey and 70% (*n* = 96) during the post-intervention survey (Table 2).

## 4. DISCUSSION

The present study demonstrated that a targeted scenario-based training intervention significantly improved ORNs’ knowledge and self-reported adherence to safe perioperative transfer practices for patients with SCI. Notable improvements were observed in the preferred use of plastic boards, compliance with the recommended four-person transfer team, and clearer identification of leadership responsibility during patient transfer. These findings underscore the value of structured educational interventions in enhancing awareness and promoting standardized patient-handling practices in perioperative settings. Importantly, these improvements were observed among a cohort of experienced ORNs, with a median OR experience of 10 years, indicating that even well-established clinical practices can be further refined through focused, scenario-based training.

Safe patient transfer is a fundamental component of evidence-based SCI management protocols, which aim to minimize unnecessary spinal motion and reduce the risk of secondary neurological injury.^13–24^ In the present study, a high baseline level of injury awareness was observed, with more than 90% of ORNs reporting that they routinely checked patient injuries before transfer. This proportion increased modestly but significantly following the intervention, suggesting reinforcement of an already well-established safety behavior. Previous investigations evaluating spinal transfer techniques have demonstrated that staffing adequacy, transfer method selection, and stabilization technique significantly influence spinal motion during patient handling.^13,16,17^ Consistent with this evidence, our results showed a significant post-intervention shift toward anesthetists directing patient transfers, with consultation increasing from 41% to 58%, while reliance on non-designated personnel decreased. This change reflects improved recognition of defined leadership roles during perioperative transfers following the training intervention. Experimental and cadaveric studies have further shown that log-roll maneuvers performed with fewer assistants generate greater cervical motion compared with transfers involving additional personnel, and that alternative stabilization techniques may reduce motion.^16,17^ Moreover, systematic evaluations indicate that commonly used log-roll techniques may produce greater spinal movement than several available alternatives, highlighting the importance of evidence-based protocols and targeted training to reduce the risk of secondary injury.^13^

Del Rossi et al.^18^ reported that transfer technique selection significantly affects spinal motion, with the lift-and-slide technique producing less lateral flexion and axial rotation than the log-roll maneuver; however, training alone did not uniformly improve performance, emphasizing individual variability in technique execution. In line with these findings, Lebel et al.^19^ objectively quantified cervical spine motion during simulated transfers using wearable inertial sensors and demonstrated that poor head–trunk alignment and inadequate synchronization during critical phases of the log-roll contributed substantially to excessive motion. Together, these studies suggest that awareness of correct technique must be supported by structured, feedback-oriented training to translate knowledge into safer clinical practice.

Earlier radiographic and experimental investigations further demonstrated that the log-roll maneuver produces greater spinal motion in unstable thoracolumbar segments than alternative transfer devices, such as backboards and scoop stretchers.^20,21^ In alignment with this evidence, awareness of the recommended four-person transfer team for patients with potential SCI increased significantly in our study, from 67% pre-intervention to 79% post-intervention, reflecting improved adherence to staffing recommendations known to reduce spinal motion. Clinical and review-based evidence has also raised concerns regarding the broader application of log rolling, particularly in patients with pelvic fractures or during trauma reception, where the maneuver may exacerbate hemorrhage or offer limited diagnostic utility due to its low sensitivity for detecting vertebral injuries.^22,23^ Collectively, these findings reinforce the importance of informed technique selection, adequate staffing, and structured education to minimize avoidable spinal motion during patient transfers.

Certain safety components, such as correct hand positioning during log rolling, demonstrated a ceiling effect, with very high baseline awareness (98%) and only marginal improvement following the intervention. Similarly, knowledge regarding the number of commands required during patient transfer remained largely unchanged. These findings suggest that some fundamental transfer principles were already well established among ORNs, whereas other domains, particularly leadership identification and staffing adequacy, were more responsive to targeted training interventions.

The choice of immobilization and transfer equipment also plays a critical role in patient safety during handling. In a randomized clinical trial, Mahshidfar et al.^24^ demonstrated that long backboards were faster to apply, provided superior immobilization, and were associated with greater patient comfort compared with vacuum mattress splints during prehospital transport of trauma patients with suspected spinal injury. Correspondingly, the present study demonstrated a significant post-intervention increase in preference for plastic boards during perioperative transfers, reinforcing alignment between evidence-based recommendations and ORNs’ reported practices. These findings support the continued use of rigid transfer devices for short-duration patient movement when rapid immobilization and controlled transfer are required. In support of this approach, a consensus statement from the Faculty of Pre-Hospital Care emphasized the principle of minimal patient handling, advocating for techniques that limit unnecessary movement and repeated transfers, thereby reinforcing the need for standardized, evidence-based transfer protocols across care settings.^25^

In the perioperative context, additional challenges arise when patients with cervical spine pathology require prone positioning for surgical intervention. A systematic review evaluating methods of prone positioning identified the “sandwich and flip” technique using specialized spinal tables as the safest approach, producing substantially less cervical motion than traditional log-roll maneuvers with manual in-line stabilization.^26^ These findings align with perioperative guidelines that emphasize careful positioning, interdisciplinary coordination, and surgeon oversight to minimize secondary neurological injury during intraoperative transfers, particularly in vulnerable populations such as pediatric patients.^27^

Despite the biomechanical advantages of specialized spinal tables, their use introduces human-factor challenges. Qualitative research examining provider perceptions of the Jackson table highlighted concerns related to workflow complexity, equipment handling, and staff safety, underscoring the importance of standardized procedures, training, and checklists to mitigate risks during patient positioning.^28^ Biomechanical validation of these concerns has been provided by cadaveric studies demonstrating that manual prone positioning generates two- to three-fold greater cervical spine motion compared with controlled mechanical rotation using a Jackson table, regardless of cervical collar use.^29^ These findings further emphasize the importance of staff awareness and training in selecting and executing the safest transfer methods.

Beyond equipment and technique selection, the way training is delivered plays a critical role in improving clinician awareness and preparedness. Evidence from early high-fidelity simulation research demonstrated that simulation-based training enhances confidence, critical thinking, and readiness for clinical practice while allowing healthcare providers to practice without exposing patients to risk.^30^ These findings support the use of scenario-based training approaches, such as those employed in the present study, to reinforce safe transfer principles and improve awareness among perioperative staff.

Overall, this study contributes to the growing body of evidence indicating that structured educational interventions can meaningfully improve healthcare workers’ awareness and self-reported adherence to safe patient transfer practices. By demonstrating statistically significant improvements across multiple domains, including leadership recognition, staffing adequacy, equipment selection, and injury awareness, this study highlights how scenario-based training can effectively translate evidence-based principles into routine perioperative practice, even among experienced nursing staff.

### 4.1 Limitations

This single-center study limits generalizability. Outcomes were based on self-reported practices rather than direct observation, introducing potential response bias. The short follow-up period did not allow for assessing long-term knowledge retention or behavior change. Only ORNs were included, excluding other perioperative staff involved in patient transfers. In addition, the questionnaire was developed using expert review and pilot testing rather than a full formal Delphi process with inter-expert agreement statistics. Future studies should incorporate objective performance assessment, multi-center designs, and longer follow-up periods.

### 4.2 Clinical recommendations

- Institutions should implement standardized protocols for perioperative transfer of patients with potential SCI.
- Regular scenario-based training should be integrated into OR competencies.
- Interdisciplinary participation involving anesthetists, surgeons, and nurses is recommended.
- Periodic audits should be conducted to ensure sustained adherence to safe transfer practices.

## 5. CONCLUSION

This survey-based pre- and post-intervention study showed that a targeted scenario-based training program significantly improved ORNs’ knowledge and self-reported adherence to safe perioperative transfer practices for patients with potential spinal injury. Implementation of structured education initiatives and standardized institutional protocols is crucial for reducing secondary SCI and improving overall patient safety in perioperative settings. To enhance these findings and further refine transfer safety practices, we call for multicenter trials and invite collaboration from institutions worldwide. A collective effort in conducting larger studies could underscore the field’s shared responsibility in advancing patient safety and galvanize stakeholders into action.

## ACKNOWLEDGEMENTS

The authors would like to thank the operating room nurses, nurse educators, and anesthetists at Hamad General Hospital for their participation and support during the training sessions and data collection process.

## CONFLICT OF INTEREST

The authors declare no conflicts of interest related to this study

## DISCLOSURE OF AI USE

The authors declare that AI-related tools were used only for grammar improvement. All scientific content, data interpretation, and final manuscript approval were performed by the authors without AI use.

## AUTHOR CONTRIBUTIONS

BW and AEM conceived and designed the study. BW, AJA, KJT, MDB, MJ, MAW, and MG were involved in data collection and implementation of the training sessions. AEM and AM performed the data analysis and interpretation. BW, AM, HAT, and AEM drafted the manuscript. All authors critically revised the manuscript, approved the final version, and agreed to be accountable for all aspects of the work.

## FUNDING

This research received no specific grant from any funding agency in the public, commercial, or not-for-profit sectors.

## Figures and Tables

**Table 1.** Demographic characteristics of operating room (OR) nurses participating in the survey (*N* = 138).

Variable	Value
Age (mean ± SD)	37.6 ± 6.2
**Gender**	
Males	44 (31.9%)
Females	94 (68.1%)
**Experience in OR**	
0–2 years	3 (2.2%)
>2–5 years	30 (21.7%)
>5–10 years	45 (32.6%)
>10 years	60 (43.5%)

**Table 2.** Comparison of pre- and post-intervention responses to survey questions on perioperative patient transfers (*N* = 138).

Survey questions	Responses	Pre-intervention	Post-intervention	*P* value
Does HMC have a policy on transferring patients with potential spine injuries?	Yes No Don’t know	90 (65.2%)32 (23.2%)16 (11.6%)	68 (49.3%)64 (46.4%)6 (4.3%)	0.001
Do you inform yourself about the patient’s potential injuries before participating in the transfer of the patient?	Yes No Don’t know	127 (92.0%)10 (7.2%)1 (0.7%)	131 (94.9%)7 (5.1%)---	0.001
Whom do you usually ask how to transfer the patient?	Staff Nurse Surgeon Anesthetist Nobody Anybody (A, B, C)	14 (10.1%)19 (13.8%)57 (41.3%)2 (1.4%)46 (33.3%)	10 (7.2%)8 (5.8%)80 (58.0%)1 (0.7%)39 (28.3%)	0.001
Which of the following is the preferred tool to be used for the transfer of patients with potential spine injury from a trolley to an operating table?	Only manpower Plastic sheet Plastic board Bed sheet and/or Blanket	3 (2.2%)9 (6.5%)122 (88.4%)4 (2.9%)	2 (1.4%)2 (1.4%)129 (93.5%)5 (3.6%)	0.001
How many staff are needed to transfer a patient (with a normal body mass index) with a potential spine injury?	2 3 4 5 6	2 (1.4%)20 (14.5%)92 (66.7%)19 (13.8%)5 (3.6%)	1 (0.7%)16 (11.6%)109 (79.0%)11 (8.0%)1 (0.7%)	0.001
Do you think that the position of hands is important in log rolling?	Yes No Don’t know	135 (97.8%)2 (1.4%)1 (0.7%)	137 (99.3%)1 (0.7%)---	0.001
How many commands are given in the transfer of patients with potential spine injury?	1 2 3 4 5 6 None	26 (18.8%)5 (3.6%)67 (48.6%)29 (21.0%)7 (5.1%)3 (2.2%)1 (0.7%)	20 (14.5%)2 (1.4%)66 (47.8%)36 (26.1%)12 (8.7%)2 (1.4%)----	0.001
Who is responsible for giving commands to initiate the transfer of the patient from a trolley to an operating table and vice versa?	Anesthesiologist Operating surgeon Whoever is on the head site of the patient Circulating nurse	101 (73.2%)5 (3.6%)31 (22.5%)1 (0.7%)	96 (69.6%)----41 (29.7%)1 (0.7%)	0.001
